# Improved Laser Ablation Method for the Production of Luminescent Carbon Particles in Liquids

**DOI:** 10.3390/ma14092365

**Published:** 2021-05-01

**Authors:** Agata Kaczmarek, Piotr Denis, Marcin Krajewski, Tomasz Mościcki, Artur Małolepszy, Jacek Hoffman

**Affiliations:** 1 Institute of Fundamental Technological Research, Polish Academy of Sciences, Pawinskiego 5B, 02-106 Warsaw, Poland; pdenis@ippt.pan.pl (P.D.); mkraj@ippt.pan.pl (M.K.); tmosc@ippt.pan.pl (T.M.); jhoffman@ippt.pan.pl (J.H.); 2 Faculty of Chemical and Process Engineering, Warsaw University of Technology, Warynskiego 1, 00-645 Warsaw, Poland; artur.malolepszy@pw.edu.pl

**Keywords:** pulsed laser ablation in liquid, carbon nanoparticles, photoluminescent particles

## Abstract

An improved method for the production of luminescent carbon nanoparticles is proposed in this work. The new method overcomes the disadvantages of commonly used approaches. It involves two-stage laser ablation in water and in aqueous solutions, where the first stage is the laser ablation of a graphite target and the second is the shredding of particles produced in the first step. The two-stage method offers the optimization of the laser pulse fluence for the performance of each process. It was found that the two-stage process of laser ablation allows producing photoluminescent carbon structures in pure water. The additional reagent may be added either in the first or second stage. The first stage performed in pure water allows avoiding the contamination of the target. Moreover, it simplifies the identification of the origin of photoluminescence. Two synthesis routes for the preparation of carbon nanoparticles by the proposed method using pure water as well as urea aqueous solution are investigated. It was found that the use of urea as a reagent results in luminescence properties similar to those obtained with other more hazardous amine-based reagents. The influence of the synthesis approach and process parameters on the structural and luminescent properties of nanoparticles is also explored in this work.

## 1. Introduction

The investigation of the mechanisms governing the photoluminescence of carbon nanoparticles (CNPs) has received substantial attention over the past decade [1,2,3,4,5,6]. In fact, it is perceived as a challenging task due to several fundamental ambiguities concerning not only the multitude of the production methods of a vast variety of different CNPs [1,2,3,4,5,6,7,8,9,10,11,12,13,14,15,16,17] but also the lack of clarity in identifying the type of nanoparticles relevant [18,19].

There is a problem associated with the separation of the fluorescent contributions of molecular fluorophores and pristine or functionalized carbon structures. This issue may arise from the fact that most studies consider the examination of carbon nanoparticles produced by bottom-up techniques, mainly based on chemical routes, such as surface passivation, synthesis from candle soot, or the hydrothermal carbonization of citric acid [1,2,4,5,12]. All of these methods result in CNPs with high photoluminescent properties. However, these synthesis approaches usually involve complex processes or need expensive starting materials, severe synthesis conditions, and result in obtaining many byproducts, which are frequently toxic. Pulsed laser ablation in liquids (PLAL) constitutes an alternative to chemical methods leading to the formation of CNPs. This method provides particles without producing byproducts, is of moderate cost, and is easier to implement than conventional approaches due to the lack of need for vacuum systems. In this approach, the solid target immersed in a liquid is subjected to laser beam irradiation. The liquid–solid interface is heated by a laser beam to temperatures reaching up to several kilokelvins, which leads to the formation of a plasma plume, confined by the surrounding liquid. Within the bubble, the pressure can reach several GPa, which enforces chemical interaction between vaporized medium and ablated species [20]. These conditions lead to the formation of many novel materials [3,6,7,8,9,10,11,13,14,15,16,18,19,20,21,22,23,24,25,26].

Although the amount of reagents used in the PLAL approach in comparison to chemical methods is rather scarce (usually only one liquid medium is involved during the process), it is still difficult to exclude the effect of fluorophore molecules on the fluorescent properties of the suspension of carbon nanoparticles. On the one hand, CNPs produced by laser ablation in water are supposed to display negligible fluorescence, but on the other hand, CNPs obtained by laser synthesis performed in other liquids (such as acetone, isopropyl alcohol, polyethylene glycol, ethylenediamine, or polyethyleneimine) exhibit complex photoluminescence even after the use of separation methods such as dialysis [3,6,9,14].

Previously, the vast majority of CNP suspensions were obtained by the direct laser ablation in liquids of target [9,10,14,15,16,17,26] or commercial carbon powder [7,8,11,23,24] only. This means that the synthesis is a one-step process, and no further postprocessing is involved. However, there are two mutually exclusive requirements in the process of nanoparticle generation by laser ablation. Firstly, during target ablation, the fluence of the laser pulse should be low, about 2–3 J/cm^2^ for a graphite target, as shown in the following section. Excessive fluence leads to the generation of fragments separated from the target by the action of recoil pressure [21,22]. Such an effect is more pronounced in the case of ablation in a liquid. Moreover, liquid splashes disturb the process.

On the other hand, low fluence leads to the production of relatively big particles. Reducing their dimensions requires values exceeding 10 J/cm^2^. The logical conclusion is to split the process into two distinct stages: preliminary target ablation with low fluence followed by the fragmentation of previously obtained suspension with high fluence.

This approach also has additional advantages, i.e., the possibility to change the liquid medium in the second stage. For instance, the first stage can be performed in pure water, and a small amount of reagent may be added only in the second step. This approach helps the identification of the photoluminescence origin of CNPs per se and the avoidance of the contamination of the target.

Herein, a novel alternative method for luminescent carbon nanoparticle production described above is presented. It allows the effective synthesis of luminescent CNPs in pure water. The usage of urea solutions as liquid media during the PLAL process has not been reported yet. Nevertheless, this nontoxic amine-based reagent may enhance the optical properties of CNPs to a degree comparable with more hazardous reagents such as ethylenediamine (EDA) or polyethyleneimine (PEI). Hence, the comparison between two synthesis routes for obtaining carbon nanoparticles (CNPs) in water and in urea aqueous solutions is carried out in this work. In addition, the influence of the synthesis approach and parameters on the structural and luminescent properties of CNPs is investigated.

## 2. Physical Background

In this section, we calculate the values of the laser pulse energy density suitable for each stage of the process. We show that appropriate values are significantly different for the ablation of the graphite target and shredding the suspension of carbon particles.

Ablation under the action of nanosecond laser pulses is thermal in nature. The absorbed energy of the pulse heats the material of the target, causing it to evaporate. The energy of the laser pulse per unit area, called fluence, is a common parameter used in the analysis. The fluence threshold value required for the rapid evaporation from the surface of a solid target can be determined from the energy balance, requiring that the laser pulse energy absorbed in an infinitesimal volume of the target must be higher or equal to the energy necessary to evaporate this volume.

(1) $$\left(1-R\right)\alpha F_{o}\exp\left(-\alpha h\right)=\rho L,$$

where *h* is the ablation depth, ρ is the material density, *L* is the latent heat of evaporation and melting, $\alpha =\frac{4\pi k}{\lambda }$ is the absorption coefficient, λ is the wavelength of laser radiation, and *R* is the surface reflectivity.

(2) $$R=\frac{{\left(n-1\right)}^{2}+k^{2}}{{\left(n+1\right)}^{2}+k^{2}},$$

where *n* and *k* are the real and imaginary parts of the complex refractive index of the target, respectively [27].

Rearranging (1) we have:

(3) $$F_{0}=\frac{\rho L\exp\left(\alpha h\right)}{\alpha \left(1-R\right)}.$$

For graphite, $\rho =2\cdot 10^{3}\;\mathrm{kg}\cdot m^{-3}$ (mean value from several brands), $L=60\;\mathrm{MJ}\cdot {\mathrm{kg}}^{-1}$ [28]. The complex refractive index data were taken from [29]. Assuming *h* = 0, we have *F*_0_ = 0.84 J cm^−2^ and *F*_0_ = 0.36 J cm^−2^ for 1064 and 355 nm, respectively. This is the threshold value for the ablation of an infinitesimally thin layer. In reality, even at the threshold, some of the finite layer is ablated. The thickness of the layer cannot be determined in such an elementary model; however, the above formula gives the correct value of the low bound of the threshold fluence. For graphite, the approximate value of *F*_0_ = 1.8–2.0 J cm^−2^ was found from both the experiment and comprehensive models [21,22] at a wavelength of 1064 as well as 355 nm, which corresponds to *h* = 35 nm at both wavelengths. For comparison with previous results, calculations were performed under vacuum. Immersing the target in water reduces its reflectivity and decreases the threshold fluence by 15%.

Increasing the fluence above the threshold increases the yield of ablation. Unfortunately, it can also cause various undesirable phenomena. All of them result in the production of large particles in the form of droplets or debris and should therefore be avoided, and the fluence should be kept at a sufficiently low level. The most common phenomenon that occurs with virtually all materials is phase explosion [28]. It takes place at high fluence, usually above 10 J cm^−2^. In the case of graphite, chipping the target due to recoil pressure is another highly detrimental phenomenon occurring at a relatively low fluence of about 6 J cm^−2^ under vacuum [21]. Immersion in a liquid doubles the recoil pressure, limiting the allowable fluence to a value of 3 J cm^−2^. As a result, the ablation of the graphite target in order to produce carbon nanoparticles in liquids can only be performed in a very narrow range of laser pulse fluence *F* = 2–3 J cm^−2^.

Similarly, the minimum value *F*_0_ of the fluence for particle evaporation can be calculated. The energy absorbed from the laser beam by a particle must be equal to the energy necessary for the evaporation of this particle.

(4) $$F_{0}AQ_{ab}=L\rho V,$$

where *V* is the particle volume, *A* is the area of particle projection on a plane perpendicular to the laser beam, and $Q_{ab}$ is the dimensionless absorption efficiency depending on the radiation wavelength, particle size, and indices of refraction (see e.g., [30]).

Rearranging (4) we have:

(5) $$F_{0}=\frac{L\rho V}{AQ_{ab}},$$

and for a special case of a spherical particle with diameter *D*:

(6) $$F_{0}=\frac{2L\rho D}{3Q_{ab}},$$

In the case of spherical particles, it is possible to have exact results for absorption efficiency [31]:

(7) $$Q_{ab}=Q_{ab}\left(m,x\right),$$

where $x=\frac{\pi Dn_{env}}{\lambda }$ is a size parameter, *n_env_* is the real refractive index of the medium (water), $m=\frac{n-jk}{n_{env}}$ is the particle refractive index relative to the medium, and *n−jk* is the complex refractive index of the particle.

**Figure 1:**
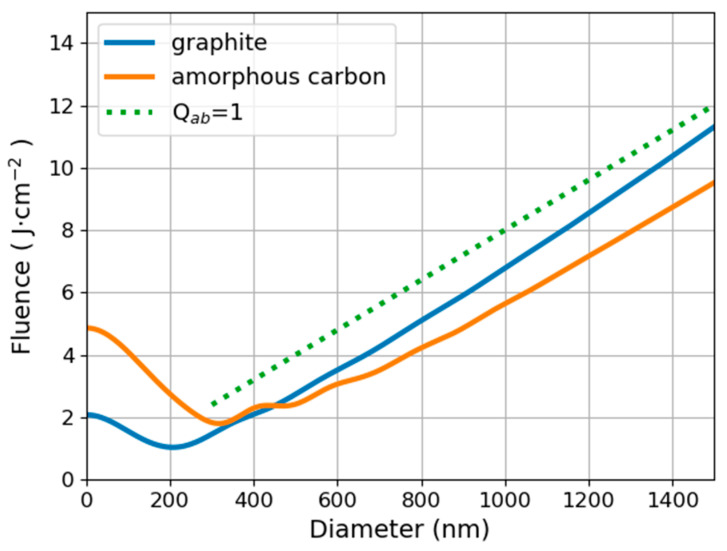
Threshold fluence necessary for the evaporation of spherical carbon particles in water by a laser beam with a wavelength of 1064 nm.

Absorption efficiency is calculated from the Mie theory of the scattering and absorption of a plane electromagnetic wave by a sphere [31]. Calculations are rather involved, but fortunately, computer programs were developed, which are freely available. For our calculations, we used the open-source program miepython [32] written in the Python programming language on the basis of [33]. The refractive index of water was taken from [34]. Because it is hard to determine the exact structure of freshly formed carbon particles, we performed calculations for both amorphous carbon and graphite using refractive indices from [35] and [29], respectively. Both results are presented in Figure 1 for a laser wavelength of 1064 nm.

As the particle size increases relative to the wavelength, its absorption efficiency tends to unity, thus $Q_{ab}≃1$ is a good approximation for large particles regardless of their optical properties. The corresponding straight line is also shown in the graph. Employing Equation (5), the results presented above may be used as an approximation even for nonspherical particles in the form of lumps. For some commercially available powders, the surface area per unit mass is specified, which makes the use of Equation (5) straightforward. It should be noted, however, that the above approximation is not valid for particles with special shapes, such as graphene flakes.

The threshold fluence increases with increasing particle size. This fact has fundamental consequences for the dynamics of the process. For instance, particles can escape the evaporation process, increasing their size by the way of coagulation. When particles too large to be evaporated are present in the suspension, they can grow using material freed from the evaporated particles. Instead of reducing the size, the process will eventually produce large particles.

From the above considerations, it is clear that the conditions suitable for ablating the target are against those necessary for the shredding of the particles. The only logical solution is to split the process into two distinct stages. The first step is the ablation of the target using low fluence, therefore avoiding the production of unwanted debris. This is followed by the second step consisting of the fragmentation of a previously obtained suspension using a fluence high enough to evaporate particles of all sizes. Failure to apply the above principles was the main reason for the widespread misconception about the poor quality of CNPs produced by laser ablation in liquids, especially in water.

## 3. Materials and Methods

### 3.1. Preparation of the CNPs Colloidal Solutions

In order to perform laser ablation, a Nd:YAG laser (981E, Quantel, Le Ulis Cedex, France) operating at 1064 nm with a 10 ns pulse duration and a repetition rate of 10 Hz was used (this is the maximum repetition frequency of this laser). Prior to experiments, the graphite target (99.997% pure, Goodfellow Cambridge Ltd., Huntingdon, England) was mechanically polished and washed several times with deionized water in order to remove surface impurities. The syntheses were in two liquid media: deionized (DI) water and aqueous urea solution (40% mas., Sigma-Aldrich Chemie GmbH, Munich, Germany).

There were two synthesis routes of CNP production. Each approach was a two-step process. In both cases, the first synthesis stage was the ablation of the target using an unfocused laser beam. The target submerged in 25 mL of liquid in a quartz beaker 40 mm in diameter was exposed to laser irradiation for 15 min with a laser power of 10 W and a beam diameter of 0.8 cm (pulse fluence = 2.0 J·cm^−2^). The resulting suspension of carbon nanoparticles was then placed in another beaker (20 mm in diameter) and exposed to further irradiation of focused laser beam (lens focal length = 750 mm, pulse fluence = 15 J·cm^−2^) with the power of 10 W for 60 min. It should be underlined that the second stage of synthesis took place without the presence of a target. During both synthesis steps, the solutions were ultrasonicated.

The synthesis conditions remained constant in both approaches. However, they differed from each other by sequence and the amount of added reagent (i.e., urea solution). In the first synthesis route (denoted as Route A), laser ablation (Step 1 (S1)) and irradiation (Step 2 (S2)) were performed directly in urea solution. In the second approach (denoted as Route B), though, laser ablation was carried out in pure deionized water, whereas laser irradiation took place in a mixture of 1 mL of urea solution and 16 mL of carbon suspension obtained from Step 1. The modification of the second approach (Route C) was also conducted. In this approach, both laser ablation and irradiation took place in pure deionized water. As it can be seen, there were five kinds of samples resulting from these synthesis schemes (B_W1 and C_W1 are identical). The used sample name consists of an indication of the synthesis approach (A, B, or C) and the number of steps. The media used are denoted as W for water and U for urea solution. Table 1 summarizes the notation and synthesis parameters of the above-mentioned routes.

### 3.2. Sample Characterization

The obtained CNP suspensions were analyzed by several techniques. The absorbance was measured with a spectrometer (Thermo Scientific Multiscan GO, Waltham, MA, USA) in the wavelength range of 200–800 nm. The instrument was zeroed to determine the zero absorbance level before the first measurement of each measurement type using a cuvette accordingly with water or urea solution. The photoluminescent spectra were obtained using a fluorescence spectrometer (FS 5, Edinburgh Instruments, Livingston, UK). The samples were prepared to obtain colloids with absorbance close to 0.1 at 350 nm. The emission spectra were collected for excitation at wavelengths ranging from 350 to 420 nm. All the optical spectra were recorded using quartz cuvettes (10 mm path length) and corrected for diluent (urea solution or water) absorption by subtracting its contribution from the recorded spectrum. Quantum yield values (QY) were assessed using quinine sulfate (in 0.1 N H_2_SO_4_ aqueous solution; QY = 55% at 350 nm) as the reference standard. The calculations were performed using the following Equation (8):

(8) $$QY_{X}=QY_{R}\times \frac{I_{X}}{I_{R}}\times \frac{A_{R}}{A_{X}}\times \frac{n_{X}^{2}}{n_{R}^{2}},$$

where *I* is the integrated fluorescence emission intensity, *n* is the refractive index, and *A* is the optical absorbance. The subscripts *X* and *R* stand for sample and reference standard, respectively [17].

Raman spectroscopy measurements were performed in a backscattering geometry using a Renishaw InVia confocal Raman spectrometer (Renishaw GmbH, Pliezhausen, Germany). Moreover, this spectrometer was equipped with a charge-coupled device (CCD) camera and two independent continuous-wave lasers with wavelengths λ = 532 nm and λ = 785 nm. Each Raman spectrum was acquired for 10 min. In order to prepare samples for Raman investigation, the produced colloid was dropped onto a silicon wafer. The solvent was then freely evaporated for 2 days in ambient conditions. The specimen prepared in such a way was placed in the measurement chamber of the Raman spectrometer.

Transmission electron microscopy (JEM-1011, JEOL GmbH, Freising, Germany) was conducted with an FEI titan instrument, operating at 300 kV, equipped with a field emission gun (FEG) and a spherical aberration corrector system (Cs-corrector) of the objective lens. In order to conduct TEM analysis, the samples were prepared by drop-casting the colloidal solutions onto a carbon-coated 300-mesh copper grid and left to evaporate at room temperature. The size distribution of nanoparticles was determined by dynamic light scattering (DLS) using the Zetasizer Nano ZS (Malvern Panalytical Inc., Westborough, MA, USA). X-ray diffraction (XRD) patterns were acquired using the D8 Discover Diffractometer Bruker AXS (Bruker, Poznań, Poland) with a CuK_α_ radiation source at a 2θ angle range of 10–50°. Suspensions were dropped on polymer substrates and dried at room temperature prior to measurements.

## 4. Results

### 4.1. Optical Measurements

#### 4.1.1. Absorbance

**Figure 2:**
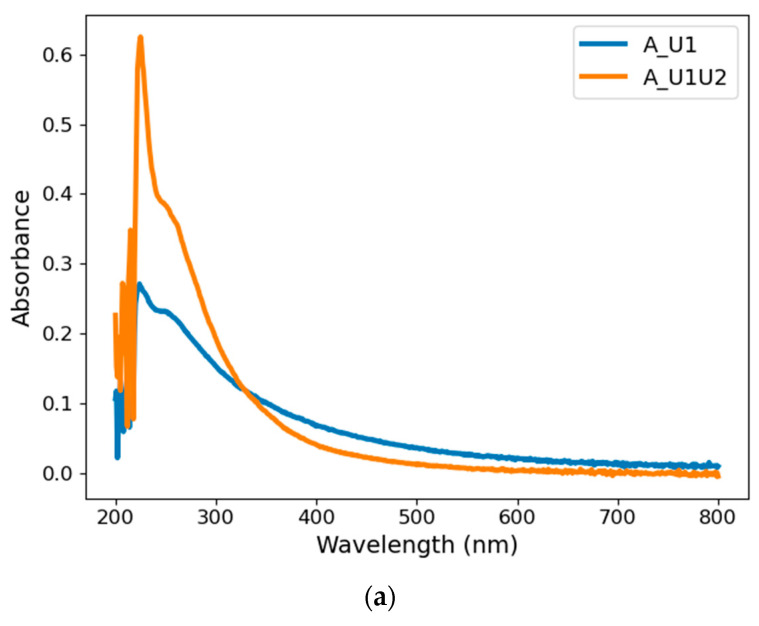

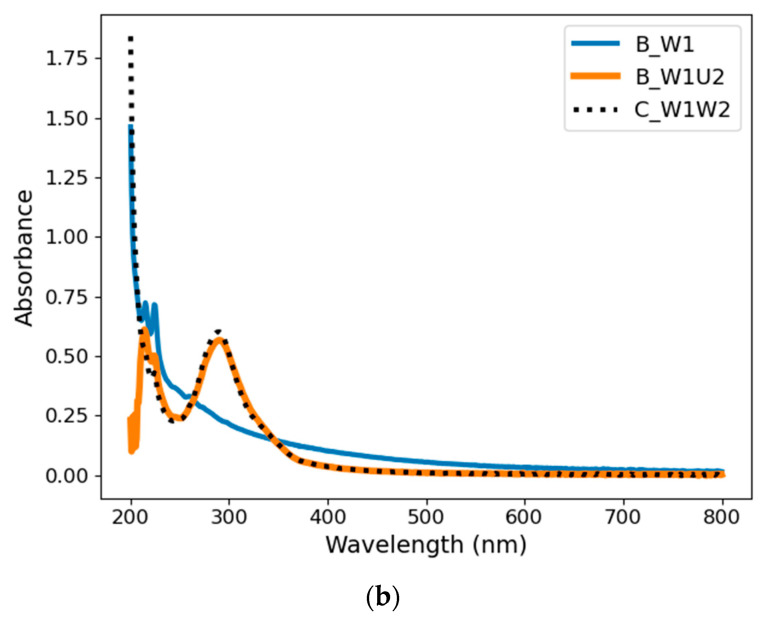
Absorbance spectra of CNPs obtained via Approaches (**a**) A and (**b**) B and C.

Figure 2 shows the optical absorption spectra of carbon nanoparticles obtained via different synthesis approaches. Figure 2a shows the absorbance after subsequent stages of Synthesis A, whereas Figure 2b compares Synthesis B and its modification, C.

As it can be seen in Figure 2a, the absorption spectra acquired from both synthesis stages (A_U1 and A_U1U2) display two absorption peaks, located at around 225 and 250 nm (the maximum of about 225 nm is distorted due to the high absorbance of the urea solution). These signals correspond to the π–π* transition of C–C bonds [1,2,3,4,5,6,8]. Although these spectra are similar, one can observe a distinctive increase in peak intensity after the second stage of Approach A accompanied by a decrease in the absorbance level at longer wavelengths. This is possibly due to fragmentation caused by the laser beam. It should be noted that after both steps of synthesis, the color of the suspensions is yellowish, indicating the presence of CNPs. Additionally, there were no significant changes in the absorption spectra of nonirradiated (reference) and laser-processed pure urea solutions. Hence, all changes of absorbance should be attributed to the presence of carbon either in the form of the target or particles during laser processing.

Absorption spectra of samples obtained from Approaches B and C (Figure 2b) differ from those from Route A. In the case of Sample B_W1, two peaks are observed in the 220–240 nm region only, which can be attributed to the presence of hydrogen-terminated polyynes [9]. This could indicate that after the first stage of synthesis, carbon nanoparticles take the form of linear carbon chains. However, the spectra of Samples B_W1U2 and C_W1W2 display an additional prominent peak around 288 nm. Our experience shows that its appearance is possible only when the fluence of the laser pulse is sufficiently high, above 15 J/cm^2^. Interestingly, the maximum absorption around 288 nm is not related to the emission of visible radiation. UV radiation is absorbed but with no luminescence results. It can be shown that the absorbance maximum is a result of Mie absorption and scattering by carbon particles when their size is sufficiently small.

In addition, there is a significant change in the color of suspensions after subsequent synthesis stages. The color of the B_W1 sample is yellowish, whereas B_W1U2 and C_W1W2 are colorless. This may demonstrate the formation of carbon structures with different morphology in both synthesis steps.

#### 4.1.2. Photoluminescence

**Figure 3:**
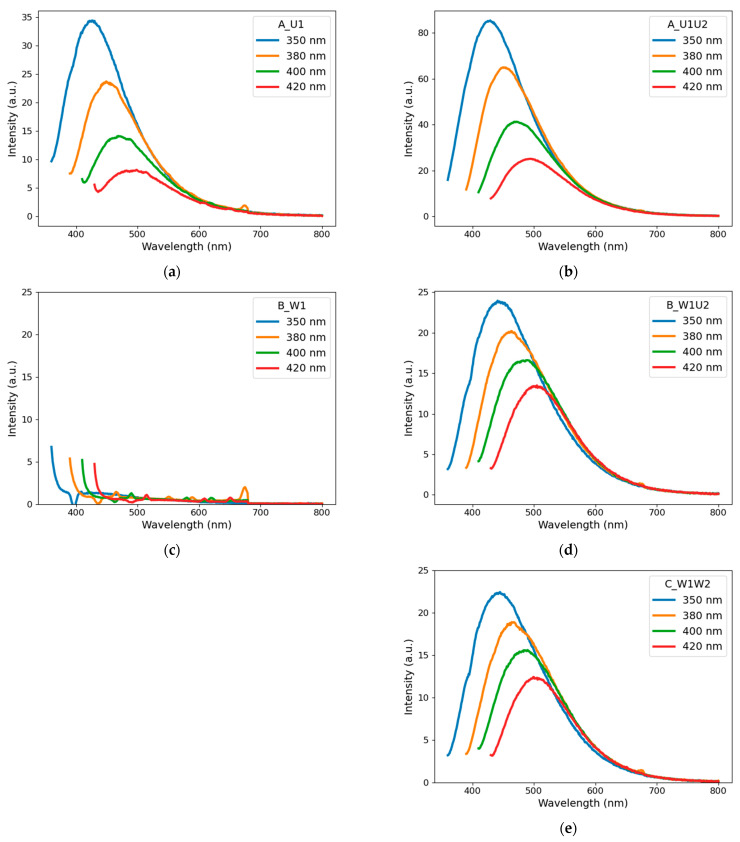
Photoluminescence spectra of carbon nanostructures obtained via Approaches (**a**) A_U1, (**b**) A_U1U2, (**c**) B_W1, (**d**) B_W1U2, and (**e**) C_W1W2. Excitation wavelengths ranged from 350 to 420 nm.

In order to explore the optical properties of CNPs, emission measurements were conducted, and the resulting emission spectra are shown in Figure 3. As it can be seen, in contrast to other samples, the photoluminescence of Sample B_W1 is negligible (Figure 3c). The signals shown in Figure 3c are mostly Raman scattering on water molecules. This may indicate that carbon nanoparticles produced in pure water do not exhibit any significant fluorescent properties per se. At the same time, in the case of other samples, the emission spectra are similar (Figure 3a,b,d,e). The following tendency can be observed that under the excitation from 350 to 420 nm the respective emission peak locations red-shift from 430 to 490 nm and that the intensity of peaks decreases with the increase in the excitation wavelength.

As it can be seen, emission peak intensities are stronger for carbon suspensions obtained via Method A than for those obtained via Route B. This can be attributed to the fact that during ablation of the target in urea solution, there is a simultaneous production of CNPs and fluorophore molecules, which affect the emission spectra [3].

Figure 3d,e shows that emission peaks intensities are similar for Samples B_W1U2 and C_W1W2. This may confirm that it is the second stage of laser processing that affects the optical properties of CNP suspensions.

It should be noted that there were no significant differences in the emission spectra of nonirradiated (reference) and laser-processed urea solutions without carbon. Hence, all changes in the emission spectra, as well as absorbance, should be attributed to the presence of carbon either in the form of target or particles during laser processing.

#### 4.1.3. Quantum Yield Determination

In order to quantitatively determine the differences between photoluminescence of samples, the quantum yield (QY) was calculated for excitation at 350 nm, and the results are summarized in Table 2.

As it can be seen from Table 2, the QY values confirm the conclusions drawn from the emission spectra. All the QY values are of the same order of magnitude as in our previous work [3] (except pure water, which was not used before). Therefore, as it can be seen, urea may enhance the optical properties of carbon nanoparticles to a degree comparable with more hazardous reagents as ethylenediamine (EDA) or polyethyleneimine (PEI). The lowest QY value of Sample B_W1 confirms the observation of the negligible fluorescent properties of CNPs produced by ablation of the graphite target in water [3,16]. Further irradiation of CNP suspension in water (Sample C_W1W2) results, however, in a significant increase in the QY value. Comparable QY values of Samples B_W1U2 and C_W1W2 are the results of the second stage of laser processing. In the case of Samples A_U1 and A_U1U2, one can observe higher QY values. It remains unclear whether the higher QYs are due to the presence of urea itself or the products of the laser’s action on it in the presence of carbon. It should be underlined that Samples BS1+2 and CS1+2 have identical within-experiment accuracy for both the absorption and emission spectra and QY values. This means that the addition of urea solution in the second stage of synthesis does not change the properties of carbon suspension obtained from the first stage. This is the second postprocessing laser stage that does change the optical properties.

### 4.2. Morphology and DLS Measurements

**Figure 4:**
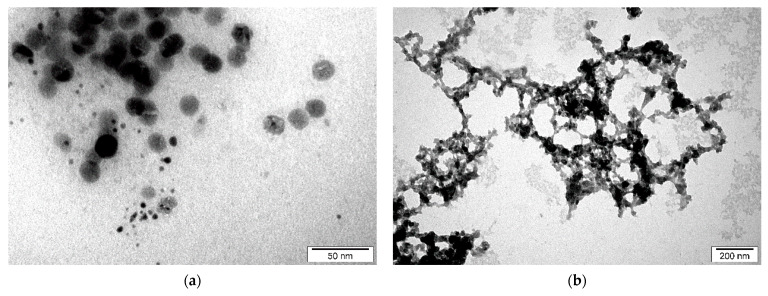
TEM micrographs of Samples (**a**) B_W1 and (**b**) C_W1W2.

The morphology and microstructure of the obtained carbon suspensions were analyzed by a transmission electron microscope (TEM). The results of the analysis are depicted in Figure 4. It should be noted that due to the crystallization of the urea solution, it was difficult to obtain clear images of CNPs for Samples A_U1, A_U1U2, and B_W1U2.

Figure 4a presents spherical CNPs produced in water (Sample B_W1). The obtained CNPs display a wide size distribution. The sample contains both smaller (~5 nm) and larger (~20 nm) structures. As it can be seen from Figure 4b (Sample C_W1W2), further irradiation of the carbon suspension in water leads to the formation of irregular structures.

**Figure 5:**
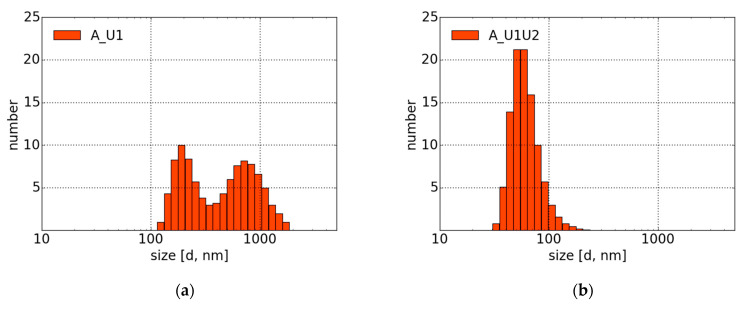

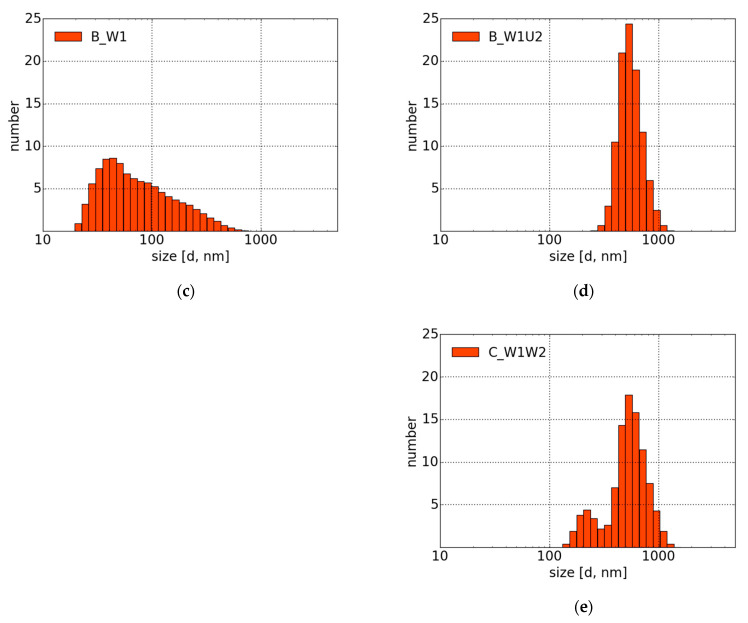
Size distributions of Samples (**a**) A_U1, (**b**) A_U1U2, (**c**) B_W1, (**d**) B_W1U2, and (**e**) C_W1W2. The mean standard deviation for all presented graphs was less than 9%.

The comparison of size distributions of Samples A_U1 and A_U1U2 (Figure 5a,b) shows that the second step of synthesis causes size reduction of particles and change in the distribution type (from bimodal in A_U1 to unimodal in A_U1U2). It means that further irradiation of samples in urea solution leads to the formation of homogeneous colloids with smaller particles.

In contrast to Approach A, further laser irradiation of carbon nanoparticles in water (Sample B_W1 versus C_W1W2) does not result in a decreased size of CNPs (Figure 5c,e). Oppositely, second-step laser processing causes the formation of apparently larger structures. Moreover, one can observe a change from a wide and unimodal (Sample B_W1) to bimodal and narrow size distribution (Sample C_W1W2). A similar tendency is observed while comparing Samples B_W1 and B_W1U2 (Figure 5c,d). However, the addition of urea solution in the second step (B_W1U2) leads to the unimodal size distribution of particles.

It is possible that the second-step laser irradiation of samples in water promotes the formation of particles with morphologies different from those obtained during laser processing of samples in urea. These findings are consistent with observations of absorption and emission spectra.

### 4.3. Structural Characterization

#### 4.3.1. X-ray Diffraction

**Figure 6:**
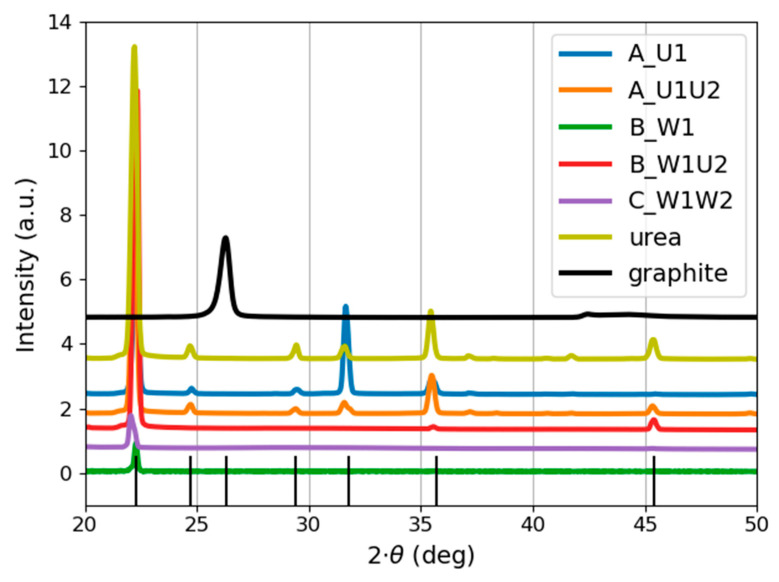
Diffraction patterns of the carbon nanostructures, reference sample (urea solution), and graphite target. The B_W1 signal was multiplied by 10. Peak positions are indicated by black vertical lines.

**Figure 7:**
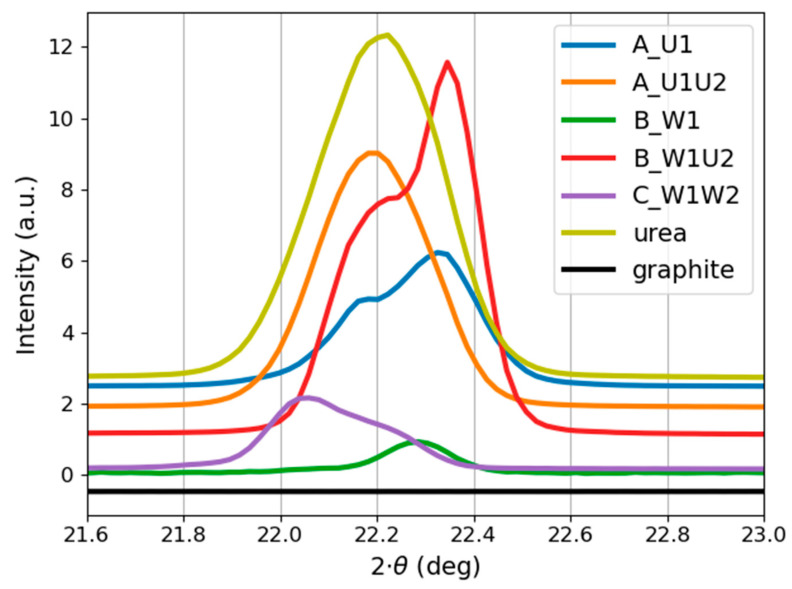
Comparison of 22° peak appearance for the carbon nanostructures, urea solution, and graphite target. The B_W1 signal was multiplied by 10.

The crystalline structure of the produced carbon structures was investigated using the XRD technique. The obtained diffraction patterns are shown in Figure 6 and Figure 7.

Figure 6 illustrates the presence of crystalline phases in all samples. Although the occurrence of coexisting amorphous phases should not be eliminated, sharp and noise-free diffraction patterns were obtained from a small amount of the material. This can indicate the presence of a significant fraction of crystalline material.

Even though the graphite target was ablated, its structure is not reflected in the XRD patterns of the obtained nanoparticles. It is especially noticeable in the case of samples produced in pure water (Samples B_W1 and C_W1W2).

In all samples, the location of the most pronounced peak is set around 22°. This could possibly suggest the presence of urea in all samples due to contamination of the target. However, it should be noted that both synthesis approaches (i.e., both target ablations) were conducted using two different targets in order to exclude the possibility of contaminating the target.

As it can be seen in Figure 6, Samples A_U1 and A_U1U2 resemble the structure of the urea solution. The peak positions are overlapping, which makes it difficult to distinguish whether the observed signals arise from carbon structures or the urea solution. This is possibly due to the fact that both samples contain a significant amount of urea solution. However, Samples B_W1, B_W1U2, and C_W1W2 have a much simpler structure (than A_U1 and A_U1U2), consisting of one (Samples B_W1 and C_W1W2) or two (Sample B_W1U2) peaks. The presence and the location of a single diffraction peak for samples produced in water implicate the similar crystalline structure of B_W1 and C_W1W2. As it can be seen, further laser processing (Sample C_W1W2) causes an increase in the peak intensity, indicating the further ordering of the obtained structures. A summary of peak locations and corresponding planes is given in Table 3.

Closer insight into the appearance of the peak located around 22° (Figure 7) demonstrates some differences between its position and form for every sample. Figure 7 shows that Sample A_U1U2 and the urea solution display a single peak located around 22.2°, whereas Samples A_U1 and B_W1U2 have a double peak-one of them is located around 22.2° and the other around 22.4°. This can indicate the involvement of two crystallographic planes, (101) and (110), respectively.

#### 4.3.2. Raman and Infrared Emission Spectra

**Figure 8:**
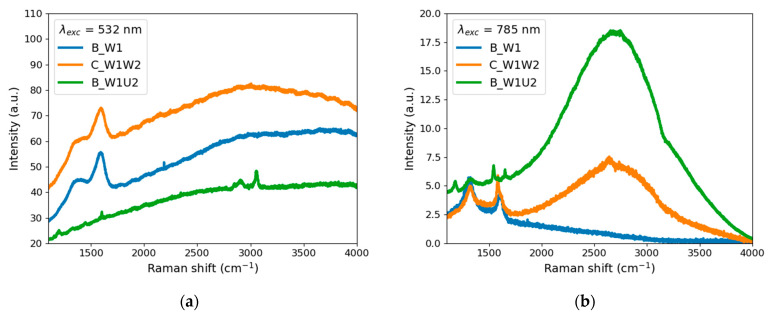
Raman spectra of Samples B_W1, C_W1W2, and B_W1U2 excited at (**a**) 532 nm (the B_W1U2 signal was multiplied by 2) and (**b**) 785 nm (the B_W1U2 signal was divided by 5).

Figure 8a,b presents the Raman spectra of Samples B_W1, B_W1U2, and C_W1W2 excited with green (532 nm) and red (785 nm) lasers, respectively.

The signal in both figures is dominated by unwanted fluorescence, even though the spectra were recorded using two different excitation wavelengths. Therefore, only repeating bands in both spectra can be attributed to Raman scattering.

As it can be observed in Figure 8, all spectra display signals attributed to representative Raman scattering for carbon structures. It is possible to identify D bands at 1360 and 1395 cm^−^^1^ and G bands at 1580 and 1589 cm^−^^1^ for B_W1 and C_W1W2, respectively. For Sample B_W1U2, small sharp peaks are observed at 1589, 2913, and 3055 cm^−^^1^ (Figure 8a); however, they are overshadowed by a strong fluorescence background.

Apart from Raman signals, it is worth comparing the infrared emission spectra in Figure 8. The most interesting is Sample B_W1U2 (blue line), for which the first step was performed in pure water and the second with urea addition. When excited with a green laser, its fluorescence is the weakest, but when excited by a red laser, it is the strongest from all samples.

It is also worth noting that Sample C_W1W2 (both synthesis steps were made in pure water) exhibits considerable fluorescence when excited by a green or red laser. It should be emphasized that the fluorescence spectra excited by the green laser are wide, and their flat maximum lies around 3000–4000 cm^−^^1^. In the case of the red laser excitation, the fluorescence spectra are narrower with a well-defined maximum at 2700 cm^−^^1^. It cannot be ruled out that the optical activity of the obtained particles is higher in infrared light than in the visible region.

The similar behavior of Samples B_W1U2 and C_W1W2 excited by a green or red laser confirms the earlier observation that second-stage laser processing influences the optical properties. The addition of urea solution (Sample B_W1U2) causes the enhancement of the effect induced by laser irradiation.

Finally, it was impossible to acquire signals coming from carbon for Samples A_U1 and A_U1U2 due to the predominance of the crystalline urea signals.

## 5. Discussion

The differences in outcomes of the first- and second-stage effects are due to the fact that the physical conditions during the first and second stages are substantially different. In the first stage, during ablation, an energy of about 1 J is absorbed on the target surface with a diameter of a few millimeters. This leads to the formation of a plasma bubble. The initial plasma temperature and pressure are very high, 25,000 K and 1.5 GPa, respectively. These values then decrease quite quickly as the bubble expands. Nevertheless, even after microsecond times, the temperature in the bubble is still around 5000 K [20]. Under the conditions described, various chemical reactions can occur. Some of them activate carbon particles, and others produce fluorophores.

During the second stage, the energy of the laser pulse is absorbed by micro- and nanoparticles. The amount of energy absorbed by a particle is of the microjoule order. The resulting plasma bubbles are tiny and very short-lived, and chemical reactions less abundant. On the other hand, during the second stage of production, particles generated in the first step can be completely evaporated, provided the fluence is sufficiently high. The de novo synthesis of carbon structures using freed carbon atoms then occurs. The resulting carbon particles can be completely different from the ablation products of the target.

XRD patterns of the samples presented in Figure 6 and Figure 7 do not resemble the structure of a graphite target. Indeed, the location of the main peak for carbon particles in pure water (Samples B_W1 and C_W1W2) varies from that of the target. Nanoparticles obtained during laser ablation are considered to be highly dependent on the initial states of the precursor (e.g., solid target and/or dispersion in liquid medium) [36]. Nevertheless, the presented results show that using high fluence values (exceeding 10 J cm^−2^) enables the creation of structures disparate from the initial material.

The particles produced in the first stage of Path C (particle production in pure water) show no measurable photoluminescence (Sample C_W1 = B_W1). The signals shown in Figure 3c are mostly Raman scattering on water molecules. Only after the second step does the photoluminescence appear, as in Figure 3e. The quantum yield is 0.27% for the excitation wavelength of 350 nm. It is not negligible, especially because, in this case, the presence of fluorophores can rather be excluded. Significant infrared emission was also observed as a side effect of Raman scattering measurements.

TEM images show the form of the carbon materials. From the first step (Figure 4a), there are spherical particles with a wide size range. The second stage brings a radical change. In Figure 4b, there are visible elaborate formations made of carbon structures. Their overall sizes can even exceed a micrometer. However, as a result of the hierarchical structure of the formations, one of the dimensions of the structures remains so small that photoluminescence is possible. The above observations are consistent with the results of DLS measurements (Figure 5c,e), which show the apparent increase in particles due to their laser treatment. However, despite an apparently large size, no sedimentation is observed even after many weeks.

The addition of urea in the second step does not change the optical properties of the particles. Absorbance, photoluminescence spectra, and quantum yield for B_W1U2 are the same as for C_W1W2. Hence, it follows that the main factor affecting the optical properties is changes in the carbon particles themselves during the second stage of laser treatment. Unfortunately, the presence of urea causes difficulties in the observation of clear TEM images. The DLS spectra for Samples B_W1U2 and C_W1W2 differ slightly. These differences are not significant, as DLS signals for both samples show a large variation over time.

The situation is quite different when urea is added during the ablation of the target. There is significant photoluminescence right after the first stage. The second stage increases this almost threefold. The maximum of the emission spectrum is shifted toward shorter wavelengths compared to Approach B, which may indicate a different origin of fluorescence. In the case of Approach A, it is impossible to exclude the production of fluorophores, which may be responsible for a considerable part of the emission. The presence of urea hinders the ability to obtain clear TEM images, but the DLS spectrum of A_U1 is wide, with particle sizes in the 100–1200 nm range. Only after the second stage are particle sizes are below 100 nm. Unlike Approaches B or C, the presence of urea from the outset prevents the development of formations such as those in water.

## 6. Conclusions

This paper identifies the root causes of difficulties in the synthesis of photoluminescent carbon NPs in water. These are conflicting requirements for laser pulse fluence values suitable for the target ablation and refinement of the produced particles. The proposed two-stage method resolves this contradiction and enables the optimization of the fluence for each stage. The corresponding values were calculated in a simple model. In reality, a range of intensity values are present in the laser beam; hence, estimated values are only indicative. The practical conclusions from the calculations are to have during the first step a fluence as low as possible (and still have ablation) and a sufficiently high fluence for the evaporation of particles of all sizes during the second stage. The presented results show that using high fluence values (exceeding 10 J·cm^−2^) enables the creation of structures disparate from the structure of the initial material. This is evidence of the complete evaporation of the particles during the second phase. The use of a two-step method enables the synthesis of luminescent CNPs in pure water. Moreover, a two-step method with the use of urea aqueous solution enables the synthesis of CNPs with photoluminescence properties similar to those obtained with other more hazardous amine-based reagents.

## Figures and Tables

**Table 1:** Notation and synthesis parameters of carbon nanoparticle suspensions.

Synthesis Approach	Laser Ablation (Step 1) Parameters	Laser Irradiation (Step 2) Parameters	Sample Name
A	Target Unfocused laser beam 25 mL of aqueous urea solution 15 min processing time 10 W laser power	-	A_U1
	Target Unfocused Laser Beam 25 mL of aqueous urea solution 15 min 10 W	No target Focused laser beam 16 mL of CNPs from S1 60 min 10 W	A_U1U2
B	Target Unfocused laser beam 25 mL of water 15 min 10 W	-	B_W1
	Target Unfocused laser beam 25 mL of water 15 min 10 W	No target Focused laser beam 1 mL of urea solution + 16 mL of CNPs from S1 60 min 10 W	B_W1U2
C	Target Unfocused laser beam 25 mL of water 15 min 10 W	-	C_W1 = B_W1
	Target Unfocused laser beam 25 mL of water 15 min 10 W	No target Focused laser beam 16 mL of CNPs from S1 60 min 10 W	C_W1W2

**Table 2:** Quantum yield values calculated for samples obtained via Routes A, B, and C.

Sample Name	QY Value (%)
A_U1	0.46
A_U1U2	1.46
B_W1	0.02
B_W1U2	0.27
C_W1W2	0.28

**Table 3:** Peak locations with corresponding planes for carbon structures and urea solution.

Peak Location (°)	Corresponding Plane	Sample Name
~22	(110) or (101)	all samples and the reference
24.7	(101)	reference, A_U1, A_U1U2
29.4	(111)	reference, A_U1, A_U1U2
31.8	(020)	reference, A_U1, A_U1U2
35.7	(120)	reference, A_U1, A_U1U2
45	(220)	reference, A_U1U2, B_W1U2

## Data Availability

The data presented in this study are available on request from the corresponding author.
